# Understanding the Role of *Prevotella* Genus in the Digestion of Lignocellulose and Other Substrates in Vietnamese Native Goats’ Rumen by Metagenomic Deep Sequencing

**DOI:** 10.3390/ani11113257

**Published:** 2021-11-14

**Authors:** Trong-Khoa Dao, Thi-Huyen Do, Ngoc-Giang Le, Hong-Duong Nguyen, Thi-Quy Nguyen, Thi-Thu-Hong Le, Nam-Hai Truong

**Affiliations:** 1 Institute of Biotechnology, Vietnam Academy of Science and Technology, 18-Hoang Quoc Viet, Cau Giay, Ha Noi 10000, Vietnam; khoadt2103@gmail.com (T.-K.D.); dohuyen@ibt.ac.vn (T.-H.D.); giangln@gmail.com (N.-G.L.); duongnguyen96uet@gmail.com (H.-D.N.); quynhungcuong@yahoo.com (T.-Q.N.); lethuhong@ibt.ac.vn (T.-T.-H.L.); 2 Graduate University of Science and Technology, Vietnam Academy of Science and Technology, 18-Hoang Quoc Viet, Cau Giay, Ha Noi 10000, Vietnam

**Keywords:** Illumina *de novo* deep sequencing, lignocellulose digestion, *Prevotella*, starch digestion, Vietnamese goats’ rumen

## Abstract

**Simple Summary:**

*Prevotella* is an abundant genus which plays an important role for lignocellulose digestion in goat rumen and is significant to the yield and quality of milk and meat in cows. In a previous study, we sequenced bacterial metagenome from Vietnamese native goat rumen to get 8.4 GB clean data and found very diverse genes related to lignocellulose degradation. However, due to the limitation in the metagenomic size, low amount of complete lignocellulase genes, and high bacterial diversity, further analysis was restricted. In this study, metagenomic deep sequencing was used to obtain 48.66 GB of reliable data, thus some lignocellulolytic enzymes were first seen and a picture of bacterial enzymes involved in lignocellulose digestion in the goat rumen was drawn. The loci for galactan-, xylan-, and arabinan-processing in *Prevotella* were observed for the first time. We confirm that *Prevotella* plays pivotal role for hemicellulose digestion and significantly participates in starch, cellulose, hemicellulose, and pectin conversion in the goat rumen. A gene coding endoxylanase was expressed in *E. coli*. The recombinant enzyme was tolerant with some salts, detergents, and had high activity, thus is a good candidate for lignocellulose processing and as an animal feed food additive to effectively improve xylooligosaccharide production.

**Abstract:**

Bacteria in rumen play pivotal roles in the digestion of nutrients to support energy for the host. In this study, metagenomic deep sequencing of bacterial metagenome extracted from the goats’ rumen generated 48.66 GB of data with 3,411,867 contigs and 5,367,270 genes. The genes were mainly functionally annotated by Kyoto Encyclopedia of Genes and Genomes (KEGG) Carbohydrate-Active enZYmes (CAZy), and HMMER database, and taxonomically classified by MEGAN. As a result, 65,554 genes encoding for 30 enzymes/proteins related to lignocellulose conversion were exploited, in which nine enzymes were seen for the first time in goat rumen. *Prevotella* was the most abundant genus, contributing 30% hemicellulases and 36% enzymes/proteins for lignocellulose pretreatment, and supporting 98.8% of feruloyl esterases and 71.7% acetylxylan esterases. In addition, 18 of the 22 most lignocellulose digesting- potential contigs belonged to *Prevotella.* Besides, *Prevotella* possessed many genes coding for amylolytic enzymes. One gene encoding for endoxylanase was successfully expressed in *E. coli*. The recombinant enzyme had high Vmax, was tolerant to some salts and detergents, worked better at pH 5.5–6.5, temperature 40–50 °C, and was capable to be used in practices. Based on these findings, we confirm that *Prevotella* plays a pivotal role for hemicellulose digestion and significantly participates in starch, cellulose, hemicellulose, and pectin digestion in the goat rumen.

## 1. Introduction

Microorganism composition in the rumen of ruminants is diverse and plays essential roles in the digestion of complex of fibrous substrates, mostly lignocellulose, into fermentable sugars [1,2]. The sugars ultimately were fermented by bacteria to produce volatile fatty acids (VFA). VFAs are the major energy source (70%) for ruminants. The majority (90%) of VFAs are acetate, butyrate, and propionate that are absorbed into the blood through the rumen wall and immigrate into the liver. In the liver, VFAs are converted into other energies for synthesis of, e.g., lipids, lipoproteins, and lactose, for body growth and development, pregnancy, and milk production [3]. Bacteria belonging to *Ruminococcus* and *Fibrobacter* genera are considered to be the most important efficient cellulose decomposers because they possess a complex of multidomain enzymes with a wide variety of catalytic activities that are assembled into cellulosomes attached to the cell membrane or distributed on the cell membrane, periplasm, or intracellular compartment [4,5,6,7]. The genus *Prevotella* is found to be effective in the digestion of xylans and pectins because it has gene clusters coding for many kinds of enzymes especially xylanase GH10, beta-xylosidase GH43, hydrolyzing xylans, and pectins [8]. In addition, in rumens of animals, *Prevotella* is emerging as a very important genus related to the milk quality of high fat protein in cows [9,10]. In goat rumen, *Prevotella* was found to be the most abundant genus [1,11], and plays an important role in host adaption to the plant fiber diet [12,13]. *Prevotella* was also shown to increase starch hydrolysis, but seemed to be not related to protein digestion in goat rumen [14]. *Prevotella* is also known as a proteolytic bacteria that produces cystein protease and collagenase [15,16]. Elucidating the role of *Prevotella* in nutrient digestion is important to modulate the environment and microbial communities to improve meat, milk quality, and yield.

In Vietnam, the native goat breed Co and hybrid breed Bach Thao, bred by Beetal and Jamunapari (derived from India), are very popular because of their delicious meat quality. Typically, the crude protein content in the meat of goat Co is high, and rich in essential amino acids [17]. The meat yield and percentage of pure meat of Bach Thao goats are higher than in Co goats; however, the quality of meat expressed through the meat pH, color, and water holding capacity is the same [18]. Vietnam is a tropical country with a developed agriculture, and goats are often grazed naturally with small herd sizes and low nutritional requirements. The diet of both goat lines Co and Bach Thao that were selected for this study consisted of a variety of grasses, leaves of trees in the mountains, and crop residues at night. In a previous study, rumen bacteria of five Co goats and five Bach Thao goats was extracted and sequenced on Illumina platform 2500 (BGI company, HongKong, China) [19]. The analysis of 8.4 GB metagenome of bacteria in these goats rumen identified 164,644 genes, of which 122,304 genes were functional annotated and 39,579 genes were linked to NCBI taxonomy [19]. *Prevotella* was the most abundant genus accounting for 35.3% of taxonomic genes. *Prevotella* also was found to contribute 40.16% of genes related to lignocellulose degradation [11]. Thus, we hypothesize that *Prevotella* in the goat rumen possesses very diverse enzymes and may hold key enzymes for effective hemicellulose degradation over enzymes for hydrolyzing xylans and pectins, and plays an important role for digestion of other foods. However, because of the limitation in metagenomic size (coverage of contigs to reads was 27.3%), the low abundance of complete lignocellulase genes (1531 genes), and high bacterial diverse, the analysis of *Prevotella* role and the overview picture of lignocellulose digestion in the goats’ rumen was restricted [11].

Bacteria in the genus *Prevotella* not only play pivotal roles for nutrient digestion in the rumen, but also are potential producers of novel and bifuctional endo-1,4-beta-xylanase (called endoxylanase in this study) [20,21,22]. This enzyme is very important in the industry for the conversion of regenerated biomass lignocelluose to produce bio-ethanol and many added-value products [23].

In this study, Illumina *de novo* deep sequencing was used to sequence the bacterial DNA metagenome extracted from the rumen of Co and Bach Thao goats to generate 48.66 GB. All the genes encoding for proteins/enzymes related to the lignocellulose digestion were analyzed to provide an overview picture of lignocellose digestion in the goats’ rumen. The role of *Prevotella* in the lignocellulose degradation was clarified by the taxonomic classification of the lignocellulase genes and identification of the lignocellulose utilization regions in some potential contigs. Then all the genes belonging to *Prevotella* were retrieved and functionally annotated to understand the contribution of *Prevotella* in other nutrients’ digestion. Finally, a gene encoding for endoxylanase with high homology to xylanase from *Prevotella* was expressed and characterized.

## 2. Materials and Methods

### 2.1. Materials

In our previous study, conventional Illumina *de novo* sequencing was used to sequence bacterial metagenome extracted from the rumen of native goats Co (five animals) and Bach Thao (five animals) that were fed with high diet of lignocellulose [19]. From the obtained 8.4 GB metagenome, 164,644 genes (or in other word, open reading frames) were identified and 122,304 genes were functionally annotated. From which, 39,501 genes from bacteria were classified into 517 genera and 1634 species [19]. *Prevotella* (13,967 genes) was the most abundant genus accounting for 35.4% taxonomic genes in the bacterial community and also was the most dominant genus in the bacterial community involved in lignocellulose hydrolysis [11].

In this study, the bacterial metagenomic DNA extracted from the goats’ rumen in the previous study was deeply sequenced to generate 48.66 GB of metagenomic data. The genes from the data were predicted and functional annotated to get an overview picture of enzymes/proteins involved in in lignocellulose degradation and to assess the role of *Prevotella* genus in the digestion of lignocellulose and other nutrients in the Vietnamese goats’ rumen through the genes harbored by *Prevotella*.

### 2.2. Metagenomic Sequencing and Assembly

The bacterial metagenomic DNA was deeply sequenced by Illuminar HiSeq 2500 (Illumina, San Diego, CA, USA) to generate 392.63 million reads. The raw sequence data was filtered to obtain accurate and reliable data of 48.66 GB with Q30 reached 94.59% by removing the reads containing at least 5% “N” bases; the reads containing adapter sequences as well as the reads containing at least 50% low quality bases (Q < 20). The clean reads were then assembled *de novo* into contigs with idba and megahit software with a series of different k-mer size in parallel. The “clean reads” were aligned against the assembly by Bowtie 2 with parameters “-p8--very-sensitive-local-k 100--score-min L,0,1.2” in order to choose the most optimal k-mer size. The 3,411,867 contigs were assembled with regard to N50 of 1162 bp and coverage of 64.22%. MetaGeneMark (version 2.10, default parameters) was used to predict open reading frames (ORFs) from assembled contigs [24]. Total predicted genes (5,367,270 ORFs) were clustered using CD-hit with sequence identity ≥95% alignment coverage ≥95% [25].

### 2.3. Functional Annotation and Analysis of Proteins/Enzymes Involved in Lignocellulose Digestion in the Goats’ Rumen

All the protein sequences deduced from the genes were blasted against public databases: (i) Swiss-Prot; (ii) Kyoto Encyclopedia of Genes and Genomes (KEGG) [26]; (iii) Non-supervised Orthologous Groups (eggNOG); (iv) Cluster of Orthologous Groups (COG) [27], and (v) Carbohydrate-Active enZYmes (CAZy) database (CAZy, http://www.cazy.org accessed on 14 February 2020) and (vi) HMMER package (http://www.hmmer.org, accessed on 14 February 2020) with all threshold *E* values below 10^−5^.

### 2.4. Taxonomic Assignment

Analysis of Nr BLAST output files was performed using the MEGAN (version 4.6) [28] for taxonomic assignment. The abundance of each taxonomic rank (phylum and genus levels) was summarized in histogram that was drawn with R package. Taxonomic level correlation was drawn using Krona complement tool in excel (http://krona.sourceforge, accessed on 5 February 2020).

### 2.5. Mining Genes Coding for Enzymes/Proteins Related to Lignocellulose Degradation

Based on the obtained results from KEGG functional annotation, the genes coding for lignocellulolytic proteins/enzymes were retrieved and then combined with taxonomy together to see abundant genera related to lignocellulose digestion in the goats’ rumen. The domains of each enzyme/protein sequence were analyzed by HMMER and PFAM with all threshold *E* values below 10^−5^. The brief map of proteins/enzymes participating in lignocellulose digestion was drawn by Microsoft Excel 2010. On the other hand, the contigs harboring at least four genes encoding for lignocellulose-hydrolyzing proteins/enzymes were filtered to find gene clusters (or lignocellulose utilization loci) in the contigs and to find the most potential producer for lignocellulose digestion.

### 2.6. Analysis of Prevotella Role in Digestion of Lignocellulose and Other Nutrients in the Goats’ Rumen

The genes that were classified to belong to *Prevotella* genus were filtered into one file. To evaluate the role of *Prevotella* in lignocellulose degradation, the *Prevotella*-deriving genes encode for enzymes/proteins involved in lignocellulose degradation annotated by KEGG were identified. The remaining genes belonging to *Prevotella*, including the genes that have not been functional annotated by KEGG, were subjected into CAZy and HMMER database to find out the functional domains for understanding the role of *Prevotella* in digestion other nutrients in the goats’ rumen.

### 2.7. Endoxylanase Expression, Purification and Characterization

Endoxylanase is one of the most important enzymes hydrolyzing xylan into short xylopolysaccharides and xylobioses, resulting in the release of celluloses for cellulase access and conversion into glucoses. Additionally, several other hemicellulases further convert the endoxylanase products to xyloses. Both glucoses and xyloses are good carbon sources supplying energy for the host activities including producing high yields of milk and meat. Endoxylanases derived from *Prevotella* genus in the rumen have been emerging as valuable enzymes capable of hydrolyzing not only xylans but also the lignin–xylan complex to enhance lignocellulose conversion, as the enzymes contain two catalytic domains GH10 and CE1 [22,29]. Thus, all amino acid sequences of endoxylanases deduced from complete genes that were mined from both conventional [11] and deep Illumina sequencing data were searched for the most similar sequence in NCBI protein database by BLASTP. The enzymes that were similar to endoxylanase of *Prevotella* contained two domains, GH10 and CE1, were retrieved. The melting temperature and pH-dependence of every enzyme were predicted by online Tm predictor software (http://tm.life.nthu.edu.tw accessed on 20 July 2016 and 15 December 2019), and AcalPred (http://lin.uestc.edu.cn/server/AcalPred accessed on 20 July 2016 and 15 December 2019), respectively, based on amino acid sequence. A gene encoding thermo-alkaphilic xylanase was selected for recombinant enzyme production in *E. coli* and some enzyme properties were characterized. Three-dimensional structure and ligand binding sites of the selected enzyme were investigated by Phyre2 software [30]. In this way, a gene code [denovogenes]_5086 encoding endoxylanase GH10-CE1 [11] was chosen for *E. coli* gene expression.

The selected gene (2223 bp) contains a 5’ terminal sequence of 63 bp encoding a signal peptide and the other sequence spanning 2157 bps codes for a mature endoxylanase and a stop codon. The mature enzyme had the predicted pI of 6.27 and molecular weight of 81.75 kDa. The investigation of rare codons of *E. coli* along the gene by an online tool (https://www.genscript.com/tools/rare-codon-analysis accessed on 25 July 2016) showed that the gene was not appropriate for expression in *E. coli* with the codon adaptation index (CAI) of 0.61. However, 9% of the codons had a usage frequency lower than 10% in *E. coli*. Thus, the codons of the gene were optimized to increase CAI to 0.96%, and to substitute all the codons with frequency usage lower than 70% (Figure S1). The optimized gene encoding mature endoxylanase was synthesized by Genscript (Piscataway, NJ, USA) and cloned into pET22b(+) (Novagen) at *Nco*I and *Xho*I restriction sites. The obtained plasmid was transformed into *E. coli* BL21(DE3) (Novagen). For recombinant enzyme expression, a single-colony transformant was inoculated into 10 mL of Luria–Bertani broth supplemented with 100 µg/mL of ampicillin (LBA medium), and grown overnight at 37 °C in a rotary shaker (160 rpm). The overnight culture (0.2 mL) was transferred into 20 mL of fresh LBA and cultivated at 37 °C, 200 rpm until the optical density (OD_600_) reached 0.6–0.8. Subsequently, the cells were induced for endoxylanase expression by adding 0.1 mM IPTG and continuously grown for 5 h at 20 °C. The cells were harvested by centrifugation at 6000 rpm for 10 min at 4 °C, and suspended in water into optical density at 600 nm (OD_600_) of 10. The cells were disrupted by sonication on ice (10 pulses, 30 s each at 100 W with 20 s intermission). The soluble fraction was harvested after centrifugation at 13,000 rpm for 10 min at 4 °C. The expressed proteins in soluble and insoluble fractions were analyzed by SDS-PAGE. The recombinant endoxylanase was purified by Immobilized Metal Affinity Chromatography (IMAC) with a 5 mL Ni-charged Sepharose Fast Flow column (HisTrap; GE Healthcare). For ready to use, the column was equilibrated with 10 column volumes (CV) of 20 mM Tris buffer (Tris HCl 20 mM, NaCl 100 mM, pH 7.4) containing 20 mM imidazole. The soluble fraction was applied to the column then washed with 5 CV of the equilibrated buffer. Contaminant proteins in the column were washed with 10 CV of the buffer containing 80 mM imidazole. The target enzyme was eluted in 20 mM Tris buffer pH 7.4 containing 250 mM imidazole. The elute fractions of 2 mL were collected and were analyzed in polyacrylamide gel 12.6% by SDS-PAGE. The final purified protein solution was desalted using a PD-10 ultrafiltration column (GE Healthcare, Mississauga, ON, Canada) by gravity flow according to the manufacturer’s direction. The purity of the recombinant endoxylanase was assessed by SDS-PAGE and then by Quantity One Software (Bio-Rad, Hercules, CA, USA). The recombinant enzyme concentration was measured by Bradford method [31]. The desalted enzymes were used for determination of endoxylanase activity and some properties of the enzyme.

Endoxylanase activity was measured by incubating 0.5 mL of assay mixture containing 0.1 mL of 1% (*w*/*v*) of birchwood xylan (Sigma, Taufkirchen, Germany), 0.1 mL of PBS pH 6 5ȕ (1 L of PBS 1ȕ contained 8 g NaCl; 0.2 g KCl; 0.24 g KH_2_PO_4_; 1.42 g Na_2_HPO_4_, adjusted to pH 6) 0.1 mL of diluted enzyme, 0.2 mL deionized water for 60 min at 40 °C. Reducing sugar content released from the reaction was measured by 3,5-dinitrosalicylic acid (DNS) described by Miller [32] using glucose as standard sugar. Accordingly, the reaction was stopped by adding 0.5 mL of DNS reagent (1% DNS; 1% NaOH; 18,2% K-Na-tartrate) and boiled for 15 min. Afterward, 25 μL K-Na-tartrate 40% was added and kept for cool at room temperature and measured at 540 nm by ELISA reader. One unit of endoxylanase activity was defined as the amount of enzyme required to hydrolyze birchwood xylan to release 1 µmol reducing groups per min in the assay condition [33].

The effect of pH, temperature and some ions, detergents on the enzyme activity was evaluated in the range of pH 2.0–9.0 (pH interval 1.0) and temperature of 30–55 °C (temperature interval 5 °C) by performing the activity assay. The PBS buffer were used for pH profile determination, using HCl or NaOH to adjust to desired pH. Thermal stability was determined by pre-incubating the enzymes at temperatures of 40 °C, 50 °C, 60 °C, and 70 °C at optimal pH for 1, 2, 4, 6, and 24 min then the enzyme assays were conducted as described. The effect of metal ions (10 mM) including Mn^2+^, Fe^3+^, Ca^2+^, Mg^2+^, Cu^2+^, Ni^2+^, Zn^2+^, K^+^, and Co^2+^, and some detergents such as SDS 1%, urea 10 mM, EDTA 10 mM, 2-mercaptoethanol 10 μM, tween 80 1% and trixon-X100 1% on the enzyme activity was investigated. The enzyme activity towards 1% birchwood xylan, 1% carboxymethyl cellulose (Sigma Chemical Co., Ronkonkoma, NY, USA) was conducted in PBS buffer (pH 5.5) at 40 °C for 10 min. The reduced sugars released were measure by DNS method as described above. The enzyme activity also was checked towards substrates of 2 mM pNP-β-D-xylopyranoside (Sigma, Ronkonkoma, NY, USA) or 2 mM pNP-β-D-glucopyranoside (Sigma Chemical Co., Ronkonkoma, NY, USA) in PBS buffer (pH 5.5) at 40 °C for 10 min and detected the released p-nitrophenol (Sigma Chemical Co., USA) at OD_405_ [34]. Enzyme kinetics were determined with the birchwood xylan concentrations from 0.05 to 5% (*w*/*v*) at the optimal condition for enzyme activity.

## 3. Results

### 3.1. Metagenomic Deep Sequencing Analysis

**Figure 1:**
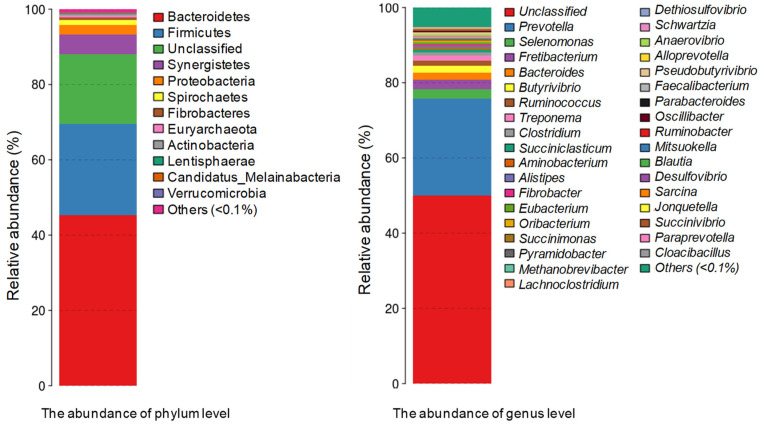
The distribution of taxonomic composition histograms at phylum and genus levels of bacterial community in Co and Bach Thao goats’ rumen.

Metagenomic deep-sequencing generated 48.66 GB of reliable data that were used to assemble into 3.411.867 contigs (mapping rate of 64.22%) with 5,367,270 genes of total 2,828,583,591 bp. Of the genes, 4,385,296 genes were functional annotated based on the deduced protein sequences alignment against Nr, Swissprot, KEGG, and eggNOG databases. In the KEGG database, a total of 2,809,791 genes were functional annotated, of which 317,154 genes (11.3%) were classified into carbohydrate metabolism (Figure S2). By MEGAN (version 4.6) software, 80.32% of the genes (4,311,093 genes of the total 5,367,270 genes) were classified into taxa. Notably, the genes belong to bacteria possessed 98.07% of the classified genes. Small portions (1.35%, 0.45%, and 0.14%) of the genes corresponded with the unknown-taxa, archaea, and eukaryote respectively. In previous study, the conventional Illumina sequencing of the sample had yielded 8.4 GB of clean data. To discriminate to deep-sequencing in this study, based on the size of data, we designated the data from conventional sequencing as “conventional data”. From the conventional sequencing, 164,644 genes had been identified and the genes belonging to bacteria accounted for 99.8% [11]. In agreement with the previous study, in deep-sequencing data, Bacteroidetes was the most abundance phylum, accounting for 45.29% of total genes or 56.71% of classified bacterial genes, followed by Firmicutes (accounted for 30.38% of the classified bacterial genes). In this research, Synergistetes and Proteobacteria accounted for 6.52% and 3.13% of all the classified genes belonging to bacteria (Figure 1). The slight variation in the abundance rates of each phylum in the bacterial community in the goats’ rumen from this data compared with previous data [11] may be the result of the Nr database being updated from 2015 to 2019 when many other phyla genes were identified. At the genus level, 49.93% of the genes were not classified. *Prevotella* was the most abundance genus accounting for 25.79% of total genes (Figure 1) (or 32.74% of the genes classified into phyla taxa), followed by *Selenomonas* (2.62%), *Fretibacterium* (2.46%), *Bacteroides* (1.86%), and *Butyrivibrio* (1.8%) (Figure 1). *Prevottella* was also the most dominant genus in goat rumen that seen in the previous investigation [11] and many other studies [1]. The *Butyrivibrio* is the most active bacteria involved in the biohydrogenation of C18 unsaturated fatty acid (FA). Species in this genus in goat rumen are complex and significant part of them are unknown species [35].

### 3.2. The Role of Prevotella in the Lignocellulose Digestion

**Figure 2:**
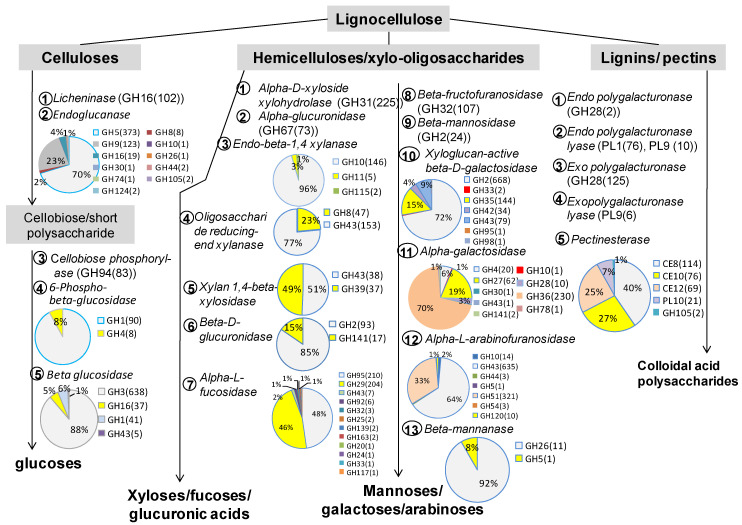
Overview picture of GHs related to lignocellulose degradation of bacteria in Co and Bach Thao goats’ rumen. Lignocellulose in the goats’ diet composed of three mains components: celluloses, hemicelluloses, and lignins/pectins. In the goats’ rumen, celluloses were digested by mainly 5 enzymes to release glucoses. Hemicelluloses were degraded by at least 13 enzymes to generate xyloses, fucoses, glucuronic acids, mannoses, galactoses, and arabinoses. Lignins/pectins were hydrolyzed by 5 dominant enzymes to produce colloidal acid polysaccharides. Some enzymes classified into only one family of GH for example licheninase. In the document, we found 102 genes coding lichenninase GH16, so abbreviated licheninase to (GH16(102)). Some enzymes had very diverse GH, for example, endo glucanase. Abbreviation: GH family (number of genes).

Based on KEGG functional annotation, a total of 65,554 genes coding for 30 enzymes/proteins related to lignocellulose conversion were exploited (Table 1). If compared to the conventional sequencing data, the size of deep sequencing data was 5.7 times the size of conventional sequencing data, the mapping rate of contigs to the reads is 2.35 times (from 27.3% to 64.22% in deep sequencing data) but the number of genes related to lignocellulose digestion increase 47-fold. Of the 65,554 genes, 8988 complete genes (Table 1) were subjected to CAZy database for investigating protein domains. As summarized in Figure 2, five important cellulases play role to converse celluloses into glucoses by two steps: firstly, licheninases, endoglucanases randomly digested celluloses into cellobioses and short polysaccharides, then three other enzymes, including cellobiose phosphorylases, phospho-beta glucosidases, and beta glucosidases, conversed the products into glucoses. All licheninases belonged to glyosyl hydrolase family 16 (GH16), almost cellobiose phosphorylases belonged to GH94 and beta glucosidases were divided into GH3, GH16, GH1, and GH4, of which the beta glucosidase GH3 accounted for 88%. The endoglucanases were very diverse, including 11 GHs; however, GH5 was the most dominant, accounting for 70% then by GH9 (23%). The conversion of hemicelluloses into mono sugars in goat rumen was conducted by at least 13 different enzymes. Of which, alpha-D-xyloside xylohydrolases, alpha-glucuronidases, endo-beta-1,4 xylanases (endoxylanase), oligosaccharide reducing end xylanases, xylan 1,4-beta xylosidases, beta-D-glucuronidases, and alpha-L fucosidases may have participated in the conversion of hemicelluloses and xylo-olygosaccharides into xyloses/fucoses and glucoronic acids.

Enzymes including beta-fructofuranosidases, beta-mannosidases, xyloglucan active beta-D galactosidases, alpha- galactosidases, alpha-L-arabinofuranosidases, and beta-mannanases were involved in the conversion of hemicelluloses/xylo-oligosaccharides into mannoses, galactoses, and arabinoses. The catalytic domains of endoxylanases were GH10, GH11, and GH115, of which endoxylanases GH10 accounted for 96%. That means GH10 plays a very important role for digesting crude hemicelluloses into short oligosaccharides in the goats’ rumen. Besides, we found very diverse enzymes involved in de-branching oligosaccharides (Figure 2). This result reflects that the goats’ diet was the complicated, diverse lignocelluloses. Lignins and pectins in the structure of lignocelluloses can be digested by five enzymes into colloidal acid polysaccharides (Figure 2). All domains of the enzymes were described more detail in the Table S1.

**Figure 3:**
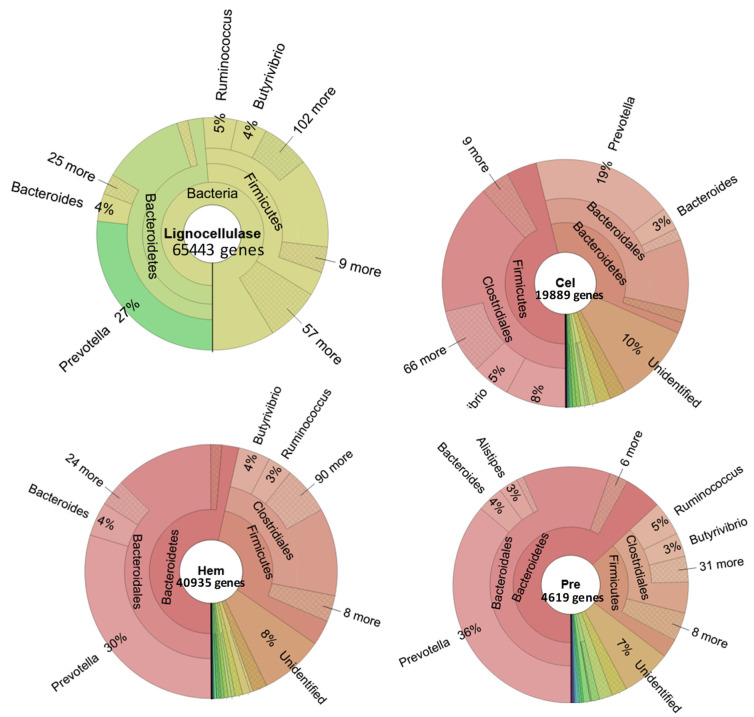
Bacterial contribution for gens coding for enzymes/proteins participated in lignocellulose digestion in Vietnamese goats’ rumen that were annotated by KEGG and taxonomic classification by MEGAN. Cel: genes coding for cellulases; Hem: genes coding for hemicellulases; Pre: genes coding for proteins/enzymes involved in lignocellulose pretreatment.

**Figure 4:**
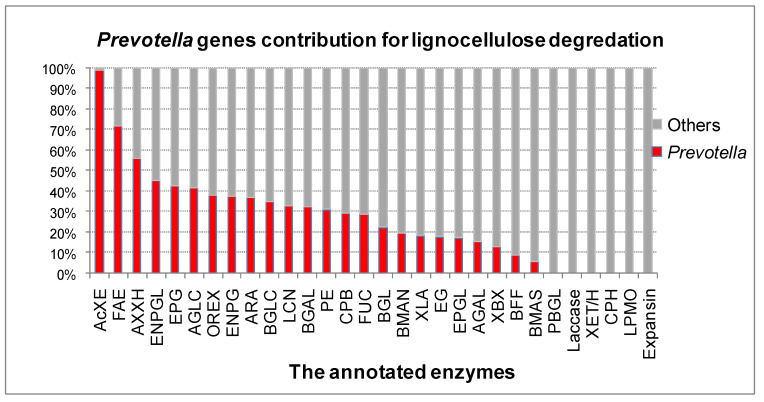
Bacterial contribution for lignocellulose digestion analyzed by metagenomic deep sequencing data of bacteria in Vietnamese goats’ rumen. The total genes included 17,495 genes that were functional annotated by KEGG, and 327 AcXE genes, 38 FAE genes, 258 EG genes were annotated by CAZy and HMMER. EG: Endoglucanase; CPB: Cellobiose phosphorylase; BGL: Beta glucosidase; PBGL: 6-Phospho-beta-glucosidase; CPH: Cellobiohydrolase; AcXE: Acetylxylan esterase; AXXH: Alpha-D-xyloside xylohydrolase; AGLC: Alpha-glucuronidase; BGLC: Beta-D-glucuronidase; BFF: Beta-fructofuranosidase; BMAS: Beta-mannosidase; XET/H: Endo-transglycosylase/hydrolase; XLA: Endo-β-1,4 xylanase; OREX: Oligosaccharide reducing-end xylanase; XBX: Xylan 1,4-beta-xylosidase; BGAL: Xyloglucan-active β-D-galactosidase; AGAL: Alpha-galactosidase; ARA: Alpha-L-arabinofuranosidase; FUC: Alpha-L-fucosidase; BMAN: Beta-mannanase: FAE: Feruloyl esterase; PE: Pectinesterase; EPG: Exopolygalacturonase; EPGL: Exopolygalacturonase lyase; ENPG: Endopolygalacturonase; ENPGL: Endopolygalacturonase lyase.

All the 65,554 genes coding for 30 enzymes/proteins involved in lignocellulose degradation in goats’ rumen were subjected to MEGAN. The result showed that 65,443 genes were classified into taxa. In genus taxon, *Prevotella* contributed 27% genes involved in lignocellulose digestion, followed by *Ruminococcus* (5%) and *Bacteroides* (4%). Remarkably, the *Prevotella* greatly supported the digestion of hemicelluloses and for pretreatment of lignocelluloses; the evidence is that *Prevotella* contributed 30% of genes for hemicellulose conversion and 36% genes for lignocellulose pretreatment (Figure 3, Table S2). Further analysis showed that *Prevotella* was not able to produce laccase, lytic polysaccharide monooxygenase, expansin or cellobiohydrolase, endo-transglycosylase/hydrolase, or phospho beta glucosidase in this document, but contributed to digestion of hemicellulose with many debranching enzymes especially acetyl xylan esterases, feruloyl esterases, and alpha-D-xyloside xylohydrolases (Figure 4). These are very important enzymes for enhancing lignocellulose conversion.

**Figure 5:**
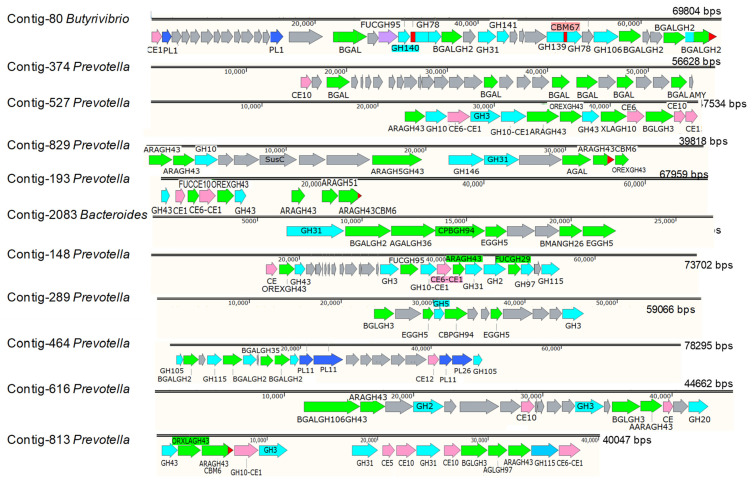
Celluloses/hemicelluloses utilization loci in some potential contigs assembled from metagenomic deep sequencing data of bacteria in goats’ rumen. CE: esterase; GH: glycosylase; PL: Polysaccharide lyase; *EG: Endoglucanase*; CBM: carbohydrate binding model; BGL: Beta glucosidase; XLA: Endo-β-1,4 xylanase; OREX: Oligosaccharide reducing-end xylanase; XBX: Xylan 1,4-beta-xylosidase; BGAL: Xyloglucan-active β-D-galactosidase; AGAL: Alpha-galactosidase; ARA: Alpha-L-arabinofuranosidase; AFUC, FUC: Alpha-L-fucosidase; BMAN: Beta-mannanase; AGL: Alpha glucosidase; AMY: amylase.

Bacteria bearing lignocellulase genes in a community can be divided into lignocellulose-digestive potential bacteria and opportunistic bacteria. The opportunistic bacteria usually produce only beta glucosidases to converse short-polysaccharide chains into glucoses for their own energy, not for the host. In contrast, potential bacteria are characterized by their ability to produce more than one enzyme to degrade lignocellulose. In the lignocellulolytic potential bacteria, the genes coding for the lignocellulolytic enzymes usually situate in certain regions in the genome. With the aim to find the potential bacterial genera, all the contigs carrying the complete genes (8900 genes) coding for proteins/enzymes related to lignocellulose digestion were retrieved. As the statistic result, 8900 genes lay in 8364 contigs, whereas 7848 contigs carried only one gene/contig. The lignocellulose-degradative potential contigs included: four contigs harbored six genes/contig, two contigs harbored five genes/contig, sixteen contigs carried four genes/contig, fifty-four contigs contained three genes/contig, and four hundred and forty contigs carried two genes/contig (Table S3). Comparison with the taxonomic classification results, we found that of the twenty-two contigs carried at least four genes/contig, eighteen contigs belonged to *Prevotella*, two contigs belonged to *Bacteroides*, one contig belonged to *Clostridium*, and one contig belonged to *Butyrivibrio*. The gene cluster related to lignocellulose digestion in each potential contig were identified and drawn by SnapGene DNA tool. The result (Figure 5) showed that almost all gene clusters related to hemicellulose degradation and specifically acted in certain substrate. For example, in the contig-80, CE1, PL1 were seen at the beginning of the contig followed by many unknown function genes, then by PL1, BGAL composed of GH43GH143GH142, FUCGH95, then four others BGALGH2 and many other GHs; all were preferred for degradation of xyloglucan in hemicellulose structure. This type of genes cluster was also observed in the contigs 374. The genes clusters for digestion of xyloarabinofuranose were seen in the contig 527, 829, 193, 148, 616, and 813 with the participation of arabinofuranosidase, oligosaccharide reducing-end xylanase, endoxylanase, esterase, alpha-galactosidase, and sometime with beta-glucosidase. One gene cluster for cellulose degradation was found in contig 289, in which genes coding for cellulases such as endo glucanase, beta-glucosidase, and cellobiose phosphorylase were observed (Figure 5).

### 3.3. The Role of Prevotella in the Digestion of Other Nutrients

For understanding the role of *Prevotella* in digestion of other nutrients in the goats’ rumen, all genes (547,751 genes) belonging to *Prevotella* genus were retrieved. A total of 17,495 genes originating from *Prevotella*, coding for enzymes/proteins related to the lignocellulose degradation that were functional annotated by KEGG, were separated. The remaining genes including the unknown functional genes were subjected into HMMER and CAZy database to get 19,416 functional annotated genes (Table S4). There were 510,840 genes which did not match the HMMER and CAZy database, meaning these genes did not relate to enzymes.

**Figure 6:**
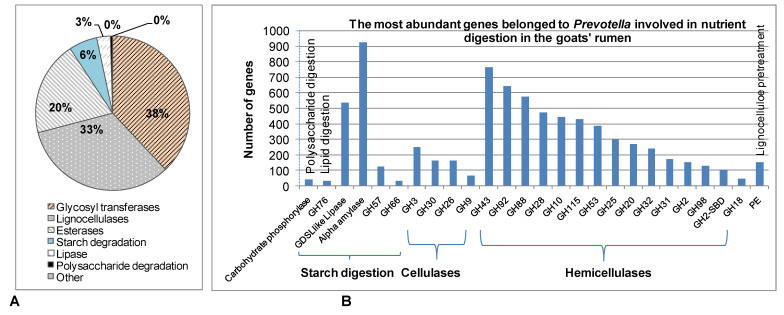
The contribution of *Prevotella* for digestion of nutrients including protein, lipid, polysaccharide, starch, and lignocellulose in Vietnamese goats’ rumen. (**A**) Percentage distribution of genes coding for enzymes involved in nutrient digestion in total 7670 genes annotated by CAZy and HMMER. (**B**) The richness enzymes related to the digestion of polysaccharide, lipid, starch, and lignocellulose that annotated by CAZy and HMMER.

The HMMER and CAZy classify genes into the GH family, but KEGG was annotated to the enzyme commission (EC) number. Thus, only some GHs families can be clarified into an EC number. By this method, of the 19,416 genes were functional annotated, there were 327 genes coding for acetylxylan esterases, 38 genes for feruloyl esterases, and 258 genes for endoglucanases GH5 that originated from *Prevotella* were identified to add into KEGG annotated lignocellulase genes (Figure 4, Table S5). The remaining genes (18,956 genes) mainly coded for glycosyl transferases (7153 genes), proteins/enzymes related to lignocellulose degradation (6709 genes), esterases (3725 genes), enzymes for hydrolyzing starch (1089 genes), lipases (537 genes), enzymes for polysaccharide degradation (87 genes), and other enzymes (116 genes) (Figure 6A, Table S6). In the glycosyl transferases group, GT2 was the most dominant with 3.562 genes then by GT1 (1925 genes). The *Prevotella*’s enzymes participated in starch conversion were mainly alpha amylase (927 genes) then by GH57, GH119, GH126, GH63, GH66, and Core2/branching enzyme. The most diverse was found in the enzymes related to lignocellulose degradation with 39 families of GH, esterase CE2 and pectin esterase (Figure 6B, Table S6).

Taking all into account, *Prevotella* in Vietnamese goats’ rumen plays a crucial, pivotal role for lignocellulose digestion and, importantly, for starch and polysaccharide degradation.

### 3.4. Expression and Characterization of Endoxylanase

Endoxylanases derived from *Prevotella* have been gained many attentions because it is highly active and stable in the presence of some detergents such as tween and SDS. From both metagenomic conventional and deep sequencing data of bacteria in the goats’ rumen, 739 genes encoding for endoxylanases annotated by KEGG were retrieved and subjected to BLASTP for the most exact similar sequences. The results showed that there were 180 amino acid sequences deduced from 180 genes matched to endoxylanase in the gene bank, of which all 108 sequences derived from *Prevotella* (Table S7). All the gene coding for sequences matched to *Prevotella* endoxylanase had a catalytic domain of GH10. Among of these, 10 genes had sequences for the accessory domain CE1, and 20 genes had sequences for an additional CBM6 domain. The endoxylanases coded by the 10 genes had a T_m_ higher than 65 °C and acidic enzymes. The gene code [denovogenes]_5086 had the lowest acidic score of 0.363/total score of 1. This means the enzyme coded by this gene may work at a light alkaline condition. Thus, we selected this gene (Figure S1) for recombinant production of endoxylanase.

**Figure 7:**
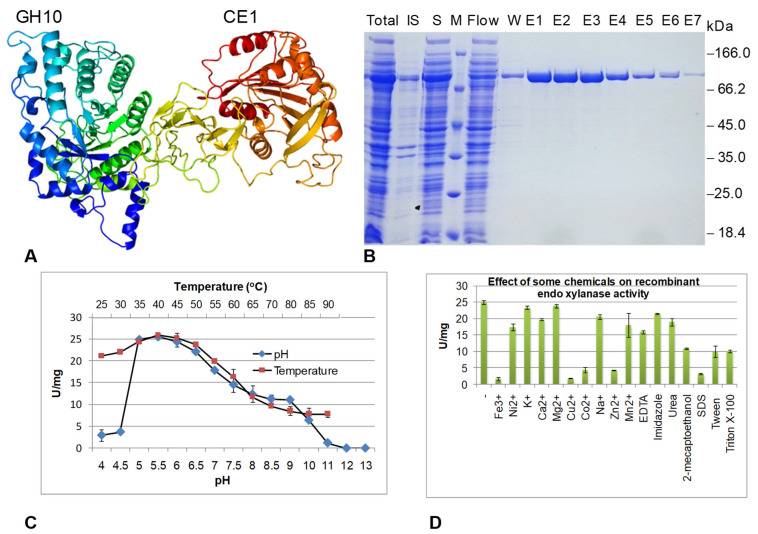
Expression, purification, and characterization of recombinant endoxylanase. (**A**) Three-dimensional structure of endoxylanase built by Phyre2; (**B**) SDS-PAGE analysis of the enzyme expression in *E. coli* Rosetta 1 strain and fractions during the enzyme purification process; (**C**) Effect of pH and temperature on recombinant endoxylanase activity towards to birchwood xylan; (**D**) Effect of some chemicals on the recombinant enzyme activity. Total: total proteins; IS: insoluble fraction; S: Soluble fraction; M: Standard protein marker (Fermentas); Flow: Unbound proteins have passed through the affinity column; W: Washed fraction; E: Eluted fraction.

By Phyre2, a three-dimensional model of the enzyme was built. Consistent with the result of the BLASTP survey of the conserved domains, the enzyme structure contained two separated domains. The catalytic domain GH10 was situated at the N terminal and CE1 at the C terminal. The sequence of GH10 was 37% similarity with alkaline thermostable endoxylanase from *Bacillus* [36] with model confidence of 100%. The CE1 domain had a 54% similarity with ferulic acid esterase [37] with the model confidence of 100% (Figure 7A).

The recombinant endoxylanase of ~81 kDa was successfully expressed in *E. coli* Rosetta 1 (Figure 7B), JM109, Origami, and poorly expressed in *E. coli* BL21, Soluble, and Rosetta 2 strains. When the cells were cultured at 37 °C, most recombinant endoxylanase was produced at an insoluble fraction. When the temperature was reduced to 20 °C for inducted cultivation, half of the enzyme was expressed in soluble form (data not shown). The suitable IPTG concentration for initiation of endoxylanase transcription then translation was 0.1 mM. The increase of IPTG from 0.1 to 1.5 did not help to increase the enzyme yield. The enzyme was produced better in the TB medium if compared to the LB medium (data not shown). Based on the Quantity One analysis, at the appropriate conditions, the recombinant endoxylanase was produced at a high level, accounting for 25–30% of total proteins from the cell mass, and ~50% of enzymes were in soluble fraction. The enzyme was successfully purified by His-tag affinity chromatography (Figure 7B). Using Quantity One software, the purity of the endoxylanase was 98%. After purification, the enzyme solution was desalted for enzyme characterization.

By the AcalPred and Tm predictor software, the endoxylanase had a Tm over 65 °C and an active pH at neutral to light alkaline. Experimental results showed that the optimal temperature for enzyme activity was 40–50 °C and the enzyme activities were reduced gradually when the temperature was increased from 50 °C to 90 °C. At 65 °C, enzyme activity was reduced by half. The recombinant endoxylanase was not an alkaline enzyme because optimal pH for enzyme action was 5.5–6.5; however, at pH8, enzyme activity remained at 50% if compared to the activity at optimal pH (Figure 7C). The recombinant enzyme was tolerant to K^+^, Mg^2+^, Na^+^, Ca^2+^, and Mn^2+^, and imidazole, urea. The enzyme had a high substrate specificity, only degrading xylan, but was not active on pNPX (a synthetic substrate for xylan beta xylosidase), pNPG (substrate for bet glucosidase), CMC, or filter paper (substrate for cellulase). The enzyme also did not hydrolyze pectin, gelatin, tributyrin, or skim milk (data not shown). The recombinant enzyme obeyed Michaelis–Menten kinetics forward to birchwood xylan. Based on a Lineweaver–Burk plot, the Km and Vmax values of endoxylanase were 14.56 mg/mL and 171.56 μmol/min/mg, respectively.

## 4. Discussion

*Prevotella* is one of nearly 40 common core genera in goat rumen, which aids in the digestion of lignocelluloses as well as in carbohydrate metabolism [1,11,38]. *Prevotella* colonization in goat rumen occurred gradually from day 14 until becoming the predominant bacteria in goat rumen in high plant fiber diets [12,38]. Up to day 110, *Prevotella* can occupy 22.37% of the genes of bacteria in goat rumen [12]. By 16S rRNA metagenomic analysis, in goats fed a dairy diet, *Prevotella* comprised 8.91% of the bacterial community, but using solid food and plants, bacteria in this genus increased by 44.02% up to 56.02% [13]. Bekele et al. investigated the diversity of bacteria and indicated that *Prevotella* included more than 50% of the bacteria in goat rumen [39]. Normally in general rumen, *Prevotella* accounts for about 42 to 60% [40]. These results are consistent with our findings on goats’ rumen, in which the goats’ diet consists of hay, a variety of grasses and leaves from mountain trees, and crop residues at night. In the metagenomic conventional sequencing data, the *Prevotella* had been found to be the most abundant genus, accounted for 35.3% of taxonomic genes [11]. By deep sequencing DNA metagenome of bacteria in Vietnamese goats’ rumen, *Prevotella* appeared to be the most abundance accounted for 32.74% genes classified into phyla taxon.

Regarding lignocellulose digestion in the goats’ rumen, although the size of deep sequencing data was 5.7 times higher than the size of normal sequencing data, the number of genes coding for proteins/enzymes involved in lignocellulose degradation was remarkably higher, up to 47.24 times, with 30 types of enzymes/proteins, of which 8.988 genes were predicted to be complete (Table 1). The enzymes hydrolyzing lignocellulose were very diverse, plentiful GHs and many types of enzymes had modular structures that contained both catalytic domains and accessory domain such as immunoglobulin-like domain (Ig), fibronectin-like domain (FN3), or carbohydrate binding domains (CBM). Some GHs were observed in a previous study by Lim et al., and Do et al. [1,11] and majority of enzymes were seen for the first time in goat rumen such as alpha-D-xylosidexylohydrolases, acetylxylan esterases, beta-fructofuranosidases, endo-transglycosylase/hydrolases, oligosaccharide reducing-end xylanases, feruloyl esterases, laccases, lytic polysaccharide monooxygenases, exopolygalacturonase lyases, and endopolygalacturonases. Interestingly, *Prevotella* supported 27% of NCBI taxonomic-affiliated genes coding for proteins/enzymes involved in lignocellulose degradation. Looking into every group, *Prevotella* contributed 19% of genes encoding for cellulases, 30% of genes encoding for hemicellulases and 38% of genes encoding for enzymes/proteins related to the lignocellulose pretreatment especially pectinesterases (Figure 3 and Figure 4). In the previous study, by conventional sequencing, we found that *Prevotella* supplied up to 40% genes for lignocellulose degradation [11]. In agreement with this result, by metagenomic analysis of bacteria-bearing genes coding for celluases in Korean goat rumen, Lim et al. also indicated that *Prevotella* species play an important role for cellulose degradation. The discovered enzymes had multiple domains in addition to the catalytic domains, which is interesting as a means to exploit new enzymes with multiple functions [1]. For example, in the normal sequencing data, we found one gene coding for endoglucanase having a modular structure consisting of X domain at the N terminal, followed by FN3 domain, and then catalytic domain GH5. In another study, we revealed that FN3 served for enzyme solubility and help for proper structure conformation [41]. The observation confirms that the big data of genes coding for proteins/enzymes involved in lignocellulose degradation are valuable for searching for novel enzymes for effective lignocellulose degradation that supports bioeconomy development.

The results obtained from further analysis showed that *Prevotella* harbored very diverse enzymes, more for hemicellulose degradation than cellulose digestion. This finding is consistent with the observation in another study on *P. bryantii* B14 [42]. Notably, many enzymes from *Prevotella* are key, pivotal enzymes for elevating lignocellulose digestion speed such as acetylxylan esterases and feruloyl esterases [43,44]. In total, 331 genes coded for acetylxylan esterases and 327 genes belonged to *Prevotella*. No genes coded for 6-phospho-beta-glucosidase, cellobiohydrolase, lytic polysaccharide monooxygenase, and expansin for cellulose digestion was found in *Prevotella* in this document (Figure 4). The obtained result confirms the pivotal role of *Prevotella* in the hemicellulose digestion in the goats’ rumen.

Supporting for the role of *Prevotella* in lignocellulose digestion, emphasizing hemicellulose degradation, 22 lignocellulose-degrading potential contigs containing at least four lignocellulase genes/contig were retrieved, of which 18 contigs belonged to *Prevotella*. The region containing a cluster of genes participating in polysaccharide degradation is designated by PUL (polysaccharide utilization loci). The PULs are usually found in digestive bacteria, representative as *Rominococcus* in human colon [45], and *Bacteroides* in rumen of animals [46,47]. However, the PULs of *Bacteroides* are complicated as they are the same as those seen in *Prevotella* in this study, because of the digestion of the most complex substrates. By analysing of genes clusters in the eleven contigs, we found nine contigs related to hemicellulose degradation, all belonging to *Prevotella*, and two contig that contributed for cellulose degradation belonging to *Bacteroides* and *Prevotella* (Figure 5). In addition, all the genes in a cluster were assembled in the same direction. Besides the main enzymes having hemicellulase activities, we also found many genes coding for enzymes belonging to different GHs, which could help in discovering the main function of the locus, and some genes were of unknown function (Figure 5). In this study, four PULs for arabinan utilization were found in contig 193, 527, 813, 829 and three PULs for galactan-processing were in contig 80, 374, 464. Some PULs related in arabinan-, xylan-, and galactan-processing were situated in contig 148, 616. The PULs for arabinan-processing were found in *Prevotella copri* in the human gut [48] but PULs for other polysaccharides degradation, including galactan-, xylan-, and arabinan-processing in *Prevotella*, were the first observation in this study.

In the investigation of the role of *Prevotella* in the goats’ rumen, we found that *Prevotella* possessed many genes coding for enzymes degrading starch and polysaccharide, and the most important enzyme for starch digestion was amylase, which is also the most abundant (Figure 6). A previous study also found that strain 25A of *Prevotella bryantiican* rapidly fermented starch and reduced the accumulation of lactate by 90% and increased amounts of succinate and propionate [49]. In this way, *Prevotella* supports much energy for the goat. The investigation of enzymes for starch and polysaccharide degradation by *Prevotella* in rumen is limited. In this study, we also found some lipases from *Prevotella*. However, *Prevotella* was known to not degrade lipids in goat rumen. *Prevotella* was reduced when feeding goats with a high lipid diet [2,50].

In conclusion, *Prevotella* is an extremely versatile genus that plays a pivotal role for hemicellulose digestion and participates in starch, cellulose, hemicellulose, and pectin digestion in the goat rumen.

Bacteria in rumen, especially in goat rumen, are very interesting for mining novel enzymes involved in lignocellulose degradation in the era of big data. Endoxylanase is one of the most important enzymes in many industries, such as for producing bioethanol, paper, and fine chemicals. Endoxylanase not only degrades xylan in hemicellulose structures for other enzymes converting xylanase products into xyloses, it also helps to expose celluloses, for cellulases attach and convert into glucoses for fermentation into final products. Endoxylanases are mainly classified into GH10 and GH11, and some in GH115. In this study, 108 amino acid sequences deduced from complete genes were high, similar to endoxylanases derived from *Prevotella*. An endoxylanase has two active domains: GH10 and CE1, which have been chosen for recombinant expression in *E. coli*. The enzyme had specificity towards birch wood xylan, but did not active in CMC, filter paper, or pectin, gelatin, tributyrin, skim milk or beta-galactosidase, or xylan beta xylosidase substrates. The recombinant enzyme was tolerance to K^+^, Mg^2+^, Na^+^, Ca^2+^, Mn^2+^, and imidazole; urea and worked better at a pH of 5.5–6.5 and temperature of 40–50 °C, as the properties of an expressed endoxylanase mined from metagenomic data of the *Nellore cattle* rumen [20] and some other sources [51,52]. The diet of farmed animals such as broilers and herbivores are regularly formulated with the inclusion of NaCl [53], thus a tolerance for NaCl will assist the enzyme for use in biotechnological sector. Endoxylanase in this study has the same size and properties and modular structure as the enzyme from *Nellore cattle* rumen; however, the enzyme from *Nellore cattle* rumen was revealed to have a bifunction to digest both xylan and p-nitrophenyl acetate (short chain esters) and Vmax of 30.959 μmol/min/mg [20]. The Vmax value of this enzyme is lower than the value of the endoxylanase in this study (171.56 μmol/min/mg). The properties of metal tolerance, especially NaCl which has an active at high Vmax in moderate temperatures, may enable enzyme application in lignocellulose biomass processing, or as an animal feed food additive for improving xylo oligosaccharide production effectively.

## 5. Conclusions

In the present study, using deep sequencing to sequence metagenomic DNA of bacteria in Vietnamese native goats’ rumen, we identified for the first time 10 kinds of enzymes coded by genes from bacteria of the goats’ rumen. *Prevotella* harbored many genes coding for key and pivotal enzymes for hemicellulose digestion and a large group of genes coding for very diverse enzymes/proteins relating to cellulose, starch, polysaccharide digestion, and lignocellulose pretreatment. These findings are valuable for guiding the investigation of the application of *Prevotella* as probiotic bacteria to improve the digestion of lignocellulose, starch, and other polysaccharides in goats, that may increase the host growth and yield of milk and meat through the conversion of VFA in the liver, and providing an adequate substrate for milk and meat synthesis. This will be investigated in future study. One endoxylanase was expressed in *E. coli*, tolerant to some salts and detergents, and had high activity, capable of the application in animal feed food additives or in lignocellulose conversion.

## Figures and Tables

**Table 1:** List of proteins/enzymes related to lignocellulose conversion that mined from metagenomic deep sequencing data and compared to metagenomic normal sequencing data [11] of bacteria in Vietnamese goats’ rumen.

Enzymes	EC Number	Abbr.	Normal Sequencing (8.4 G)	Deep Sequencing of 45 G					
				Total Genes	Lack 3’-End	Lack 5’-End	Lack Both Ends	Complete	% Complete Genes
**Cellulases**			**448**	**21,029**	**5786**	**3816**	**9091**	**2336**	**11.1**
Licheninase	3.2.1.73	LCN	1	281	49	62	68	102	36.3
Endoglucanase	3.2.1.4	EG	66	7368	1871	1406	3081	1010	13.7
Cellobiose phosphorylase	2.4.1.20	CPB	27	1439	424	268	649	98	6.8
Beta glucosidase	3.2.1.21	BGL	341	10,444	3001	1780	4702	961	9.2
6-Phospho-beta-glucosidase	3.2.1.86	PBGL	13	1281	393	274	501	113	8.8
Cellobiose dehydrogenase	1.1.99.18		0	0					
Cellobiohydrolase	3.2.1.91	CPH	0	216	48	26	90	52	24.1
**Hemicellulases**			**815**	**41,756**	**12,007**	**6658**	**17,199**	**5892**	**14.1**
Acetyl mannan esterase	3.1.1.6	AcME	0	0					
Acetylxylan esterase	3.1.1.72	AcXE	0	4	0	0	4	0	0.0
Alpha-D-xyloside xylohydrolase	3.2.1.177	AXXH	0	2833	840	460	1277	256	9.0
Alpha-glucuronidase	3.2.1.139	AGLC	37	561	155	109	224	73	13.0
Beta-D-glucuronidase	3.2.1.31	BGLC	30	888	292	127	343	126	14.2
Beta-fructofuranosidase	3.2.1.26	BFF	0	1106	368	197	427	114	10.3
Beta-mannosidase	3.2.1.25	BMAS	62	659	189	90	329	51	7.7
Endo-transglycosylase/ hydrolase	2.4.1.207	XET/H	0	2	0	1	1	0	0.0
Endo-β-1,4 xylanase	3.2.1.8	XLA	67	3400	938	634	1121	707	20.8
Oligosaccharide reducing-end xylanase	3.2.1.156	OREX	0	1213	422	191	363	237	19.5
Xylan 1,4-beta-xylosidase	3.2.1.37	XBX	10	1018	262	197	337	222	21.8
Xyloglucan-active β-D-galactosidase	3.2.1.23	BGAL	290	11,690	3326	1705	5490	1169	10.0
Alpha-galactosidase	3.2.1.22	AGAL	63	4020	1174	693	1638	515	12.8
Alpha-L- arabinofuranosidase	3.2.1.55	ARA	138	6229	1882	891	2282	1174	18.8
Alpha-L-fucosidase	3.2.1.51	FUC	55	6896	1890	1136	2663	1207	17.5
Beta-mannanase	3.2.1.78	BMAN	63	1237	269	227	700	41	3.3
**Pretreatments**			**167**	**4769**	**1382**	**782**	**1845**	**760**	**15.9**
Expansin			1	33	5	13	9	6	18.2
Feruloyl esterase	3.1.1.73	FAE	0	92	35	9	17	31	33.7
Laccases	1.10.3.2		0	9	2	2	4	1	11.1
Lignin peroxidase	1.11.1.14	LiP	0	0					
Lytic polysaccharide monooxygenase	1.14.99.56	LPMO	0	11	3	0	7	1	9.1
Manganese peroxidase	1.11.1.13	MnP	0	0					
Pectinesterase	3.1.1.11	PE	86	2525	704	483	919	419	16.6
Exopolygalacturonase	3.2.1.67	EPG	38	635	216	66	223	130	20.5
Exopolygalacturonase lyase	4.2.2.9	EPGL	0	59	8	8	37	6	10.2
Endopolygalacturonase	3.2.1.15	ENPG	0	27	4	7	14	2	7.4
Endopolygalacturonase lyase	4.2.2.2	ENPGL	42	1378	405	194	615	164	11.9

## Data Availability

The data presented in this study are available on request from the corresponding author and in the NCBI BioProject with the accession ID PRJNA749909.

## Supplementary Materials

The following are available online at https://www.mdpi.com/article/10.3390/ani11113257/s1, Figure S1: Nucleotide sequence of gene [denovogenes]_5086 and codon optimized gene encode for endoxylanase. The underlined sequence codes for signal peptide; the bold, italic sequences indicate *Nco*I, *Xho*I sites; Figure S2: KEGG pathway classification of the genes from metagenomic deep sequencing data of bacteria in the Vietnamese goats’ rumen; Table S1: Diversity of domains structure in lignocellulase deduced from complete open reading frames that were identified by CAZy and PFAM. GH: Glycosyl hydrolase; Table S2: Taxonomic assignment of the genes coding for enzymes/proteins related to lignocellulose digestion in Vietnamese goats’ rumen; Table S3: Statistical analysis the number of genes conding for proteins/enzymes related to lignocellulose degradation in each contig; Table S4: List of CAZy, HMMER—functional annotated genes belonged to *Prevotella*; Table S5: Bacteria contribution for lignocellulose digestion analyzed by metagenomic deep sequencing data of bacteria in Vietnamese goats’ rumen; Table S6: Contribution of *Prevotella* for other nutrients digestion that was analyzed from metagenomic deep sequencing data of bacteria in Vietnamese goats’ rumen; Table S7: Analysis of domains by CAZy and the most similar sequences by BLASTP of 739 complete gene coding for endoglucanase which annotated by KEGG.
