# Supplementary material for: Understanding the Role of Prevotella Genus in the Digestion of Lignocellulose and Other Substrates in Vietnamese Native Goats’ Rumen by Metagenomic Deep Sequencing
Source: Animals (Basel). 2021 Nov 14;11(11):3257. doi: 10.3390/ani11113257 (PMC8614338; doi:10.3390/ani11113257)
Supplement: Supplementary file 1 [file animals-11-03257-s001.zip › Figure S1.pdf]

Figure S1. Nucleotide sequence of gene [denovogenes]_5086 and codon optimized gene encode for endo xylanase

The underlined sequence codes for signal peptide; the bold, italic sequences indicate NcoI, XhoI sites.

```
 Original  ATG ATA AAG AAA TTA TTT GTC GGA CTG ATC CTG ACT ATG GTG GGA AGT TCG TGT GTA TTT
Optimized                                                                           CC A.G

 Original  GCA CAG TTT CGT CAG GTT GAT CCC AGA GAG AAT GTC GGC CTG AAG GAT GCC TAC AAA GGT
Optimized  .AC ... ..C ... ..A ..G ..C ..G C.T ... ..C ..T ..T ... ... ..C ..G ... ... ..C

 Original  TAT TTT ACC ATT GGT GTA GCG CTG AAC CAG CGC AAT GTA ACT GAC GAT GCC CAG AAG GCC
Optimized  ... ... ... ..C ... ..G ... ... ... ... ..T ..C ..T ..C ... ... ..G ... ... ..G

 Original  CTC GTC ATC AAG CAG TTC AAC AGT GTG ACT GCC GAG AAC GAC TGG AAG CCC GGT GAG ATT
Optimized  ..G ..G ..T ..A ..A ... ... ..C ..T ..C ..G ..A ... ... ... ... ..G ..C ... ..C

 Original  CAT CCG AAG GAA GGC GTG TGG AAT TTC GAG AAG GCT GAT AAG ATT GCT GAC TTC TGT CGT
Optimized  ..C ... ..A ... ... ... ... ..C ... ... ... ..G ..C ..A ... ..G ..T ..T ..C ...

 Original  CAG AAC GGT ATC AAG ATG CGT GGT CAT TGT CTG TGC TGG CAC TCA CAG TTT GCC GAC TGG
Optimized  ... ... ..C ... ... ... ... ... ..C ..C ... ... ... ... AGC ..A ..C ..G ... ...

 Original  ATG TTT ACC GAC AAG AAT GGC AAG CCC GTC AAG AAA GAG GTG TTC TAT CAG CGT CTG CGC
Optimized  ... ... ... ..T ... ..C ..T ..A ..G ..G ... ... ..A ..T ... ..C ... ... ... ..T

 Original  GAG CAC ATC CAT ACG GTG GTG AAC CGC TAT AAG GAT GTG GTC TAT GCC TGG GAC GTG GTC
Optimized  ... ... ..T ..C ..C ... ..T ... ..T ..C ..A ..C ... ..T ... ..G ... ..T ... ..T

 Original  AAT GAG GCT ATG GCT GAT GAC AAC CAG TTT GGT CCC CGC TTC GGA TTC GGA CGT CCG GGT
Optimized  ..C ..A ..G ... ..G ..C ..T ... ... ... ... ..G ..T ... ..T ..T ..C ... ... ...

 Original  CAG GAG CCG AGT CCG TAT CGT CAG AGC CGT CAC TTC CAG CTT TGC GGC GAC GAG TTC ATT
Optimized  ..A ... ... ..C ... ..C ... ... ... ... ... ... ..A ..G ... ..T ... ..A ..T ..C

 Original  GCC AAG GCT TTC CAG TTT GCC CGT GAG GCT GAT CCC AAT ACG TTG CTG TTC TAC AAC GAC
Optimized  ..G ... ..G ... ..A ... ..G ... ... ..G ..C ..G ..C ..C C.. ... ... ... ... ...

 Original  TAC AGC TGC GTA GAC GAG GGA AAG CGT GAG CGT ATT TAT AAT ATG GTG AAG AAG ATG AAG
Optimized  ..T ... ... ..T ..T ... ..C ... ... ..A ... ..C ..C ..C ... ... ... ..A ... ...

 Original  GAC GCT GGT GTT CCC ATC GAC GGT ATT GGT ATG CAG GGT CAC TAC AAC ATC TAC TTC CCC
Optimized  ... ..G ... ... ..G ... ..T ... ... ..C ... ... ..C ... ..T ... ... ..T ... ..G

 Original  AGC GAG GAG CAG CTT GAG AAG GCC ATT GTC CGT TTC AAG GAG ATT GTG AAG CAT ATC AAT
Optimized  ... ... ..A ..A ..G ..A ... ..G ... ..G ... ..T ... ... ..C ..T ..A ..C ... ..C

 Original  ATC ACC GAG CTC GAC CTG CGT ATG AAC AAT GAG AGC GGT GGT CAG CTG ATG TTC TCA CGT
Optimized  ..T ... ..A ..G ... ... ... ... ... ..C ... ... ... ..C ... ... ... ... AGC ...

 Original  GGT GAG GCT AAG CCC ATG CCC GCT TAC ATG TCA ACC CTG CAG ACC GAC CAG TAC GCT CGT
Optimized  ... ..A ..G ..A ..G ... ..G ..G ... ... AGC ... ... ... ... ..T ..A ..T ..G ...

 Original  CTG TTC AAG GTG TTC CGT AAG CAC GCT GAC GTC ATC GAC AAC GTG ACT TTC TGG AAT CTG
Optimized  ... ... ... ... ..T ... ..A ... ..G ... ..G ... ..T ... ..T ..C ... ... ..C ...

 Original  GGC GAC AAG GAT TCC TGG CTC GGC GTG AAC AAC CAC CCG CTG CCC TTC GAC GAG AAT TAC
Optimized  ..T ... ... ... AG. ... ..G ... ..T ... ... ... ... ... ..G ..T ... ... ..C ...

 Original  CGC CCG AAG GCT TGC TTC CGT GCC ATC CGC GAC TTC GAT CCC GCC CTC GAC AAG CGC GTG
Optimized  ..T ... ..A ..G ... ... ... ..G ..T ..T ... ..T ... ..G ..G ..G ... ... ..T ...

 Original  CCG AAG GAG GAC TTC GTC ATC AAC GAG TGG AAT CAG CCT GGT CAG GAG TGG CCG AAG GTG
Optimized  ... ..A ..A ..T ... ..T ... ... ... ... ..C ... ..G ... ..A ..A ... ... ... ...

 Original  AAC AGT GAG GGT TAT GCC CGT TTC CAG ATC GAG GCT CCC GAT GCC AAG TCG GTG ATT GTC
Optimized  ... ..C ... ..C ... ..G ... ..T ... ..T ..A ..G ..G ..C ..G ..A AGC ... ..C ..T

 Original  AGT CTC GGT CTC GGT GGT CGT GGC GGT ACG GTG CTG AAG AAG GAT GAC AAC GGC ATT TGG
Optimized  ..C ..G ... ..G ... ..C ... ..T ..C ..C ..T ... ... ..A ..C ..T ... ..T ... ...

 Original  ACA GGA ACT ACC GAG GGT CCG ATG GAT CCC GGC TTC CAC TAC TAT CAC CTG ACC ATC GAC
Optimized  ..C ..C ..C ... ... ... ... ... ..C ..G ..T ... ... ... ... ... ... ... ... ..T

 Original  GGC GCA ACT GTC AAC GAT CCC GGT ACA GGC AAC TAT TTC GGT TCG TGC CGT TGG GAG AGT
Optimized  ..T ..G ..C ..G ... ... ..G ..C ..C ... ... ..C ..T ... AGC ... ... ... ... ..C

 Original  GGT ATC GAG ATT CCC GCA CCT GAT CAG GAC TTC TAT GCT GAG CGC ACC GAT ATT CCT CAT
```

```
Optimized  ..C ... ..A ... ..G ..G ..G ..C ... ..T ... ... ..G ..A ..T ... ..C ... ..G ..C

 Original  GGC AGC ATG CAG ACG GTG AAG TTC TAT TCA CCC AGT CTT GGT AAG ATG CAG GAG GCT ACG
Optimized  ..T ... ... ..A ..C ... ... ..T ..C AGC ..G ..C ..G ..C ..A ... ... ..A ..G ..C

 Original  GTT TAT CTG CCC TAT GGC TAT GGT CAG CTC GTC GAT AAG AAG GGT AAT CTT GTG AAG GCA
Optimized  ... ... ... ..G ..C ..T ..C ..C ..A ..G ..G ... ... ..A ... ..C ..G ..T ... ..G

 Original  GGT GCC AAG GGC GTT CAG GAG CGT TAT CCT GTG CTC TAC CTG CAG CAT GGC TGG GGT GAG
Optimized  ... ..G ..A ... ..G ... ..A ... ..C ..G ..T ..G ..T ... ..A ..C ..T ... ..C ...

 Original  AAC GAG ACC AGT TGG CCC ATT CAG GGT AAG GCA GGT CTG ATC ATG GAC AAT CTG ATT GCC
Optimized  ... ..A ... ..C ... ..G ... ... ..C ... ..G ..C ... ... ... ... ..C ... ... ..G

 Original  GAC GGT AAG ATC AAA CCC TTC ATC ATC GTT ATG GCC TAT GGT CTG ACC AAC GAC TTC AAG
Optimized  ..T ..C ... ... ... ..G ... ... ..T ..G ... ..G ..C ..C ... ... ... ... ... ..A

 Original  TTC GGA ACA ATC GGT CAG TTC ACA GCT GAG GAA TTC GAG AAG GTG CTC GTC GAT GAG CTC
Optimized  ..T ..C ..C ... ..C ..A ... ..C ..G ... ... ..T ..A ... ... ..G ..T ..C ... ..G

 Original  ATT CCG TAT ATC GAC AAG AAC TTC CTG ACC AAG TCC GAC AAG TGG AAC CGC GCT ATG GCA
Optimized  ..C ... ... ..T ..T ..A ... ..T ... ... ... AG. ..T ..A ... ... ..T ..G ... ..G

 Original  GGT CTG TCG ATG GGT GGT ATG ACC ACC AAG CTC ATC ACC CTG CGC CGT CCT GAG GTG TTC
Optimized  ... ... AGC ... ... ..C ... ... ... ... ..G ... ... ... ..T ... ..G ..A ... ...

 Original  GGT TAC TGG GGC TTG CTC TCT GGC GGT CAG TAT GCA CCT GAG GAG ATC AAG GAT CCT ACG
Optimized  ... ... ... ... C.. ..G AGC ..T ..C ... ... ..G ..G ... ..A ..T ... ..C ..G ..C

 Original  GCT GTG AAG TAT ATC TTC GAG GGC TGC GGC TCT AAG GAG AAT CCT GAT GGC ATC AAC AAG
Optimized  ..G ... ..A ..C ... ..T ..A ..T ... ... AGC ..A ... ..C ..G ..C ... ..T ... ...

 Original  AGC GTT GCC GAC CTG AAA GCT GCC GGC TTC AAT GCC GAA GGT CTG ATC TCA GAG GGT ACA
Optimized  ... ... ..G ..T ... ... ..G ..G ... ... ..C ..G ..G ... ... ... AGC ..A ..C ..C

 Original  GCC CAC GAG TTC CTG ACC TGG CGC CGC TGC CTC CGT CAG ATG GCC CTG AGC CTG TTC AAG
Optimized  ..G ... ... ..T ... ... ... ..T ..T ... ..G ... ..A ... ..G ... ... ... ... ..A

 Original  TAA  -2221
Optimized  CTC GAG -2171
```
