# Supplementary material for: Understanding the Role of Prevotella Genus in the Digestion of Lignocellulose and Other Substrates in Vietnamese Native Goats’ Rumen by Metagenomic Deep Sequencing
Source: Animals (Basel). 2021 Nov 14;11(11):3257. doi: 10.3390/ani11113257 (PMC8614338; doi:10.3390/ani11113257)
Supplement: Supplementary file 1 [file animals-11-03257-s001.zip › Table S5.pdf]

Table S5. Bacteria contribution for lignocellulose digestion analyzed by metagenomic deep sequencing data of bacteria in Vietnamese goats' rumen.

Enzymes	Abbre-viation	Prevotella			Other bacteria	Total
		KEGG annotated genes	CAZy-HMMER annotated genes	Total		
Acetylxylan esterase	AcXE	0	327	327	4	**331**
Feruloyl esterase	FAE	0	38	38	15	**53**
Alpha-D-xyloside xylohydrolase	AXXH	1508		1508	1191	**2699**
Endopolygalacturonase lyase	ENPGL	608		608	745	**1353**
Exopolygalacturonase	EPG	271		271	364	**635**
Alpha-glucuronidase	AGLC	227		227	317	**544**
Oligosaccharide reducing-end xylanase	OREX	453		453	744	**1197**
Endopolygalacturonase	ENPG	9		9	15	**24**
Alpha-L-arabinofuranosidase	ARA	2258		2258	3888	**6146**
Beta-D-glucuronidase	BGLC	309		309	577	**886**
Licheninase	LCN	92		92	188	**280**
Xyloglucan-active β-D-galactosidase	BGAL	3741		3741	7848	**11589**
Pectinesterase	PE	774		774	1719	**2493**
Cellobiose phosphorylase	CPB	416		416	1014	**1430**
Alpha -L-fucosidase	FUC	1953		1953	4803	**6756**
Beta glucosidase	BGL	2221		2221	7713	**9934**
Beta-mannanase	BMAN	227		227	945	**1172**
Endo-β-1,4 xylanase	XLA	583		583	2610	**3193**
Endoglucanase	EG	948	258	1206	5592	**6798**
Exopolygalacturonase lyase	EPGL	10		10	48	**58**
Alpha-galactosidase	AGAL	620		620	3364	**3984**
Xylan 1,4-beta-xylosidase	XBX	132		132	883	**1015**
Beta-fructofuranosidase	BFF	97		97	1007	**1104**
Beta-mannosidase	BMAS	37		37	607	**644**
6-Phospho-beta-glucosidase	PBGL	1		1	1278	**1279**
Laccase	Laccase	0		0	5	**5**
Endo-transglycosylase/hydrolase	XET/H	0		0	2	**2**
Cellobiohydrolase	CPH	0		0	168	**168**
Lytic polysaccharide monooxygenase	LPMO	0		0	5	**5**
Exxpansin	Expansin	0		0	31	**31**
**Total**		**17495**	**623**	**18118**	**47690**	**65808**
