# Supplementary material for: Understanding the Role of Prevotella Genus in the Digestion of Lignocellulose and Other Substrates in Vietnamese Native Goats’ Rumen by Metagenomic Deep Sequencing
Source: Animals (Basel). 2021 Nov 14;11(11):3257. doi: 10.3390/ani11113257 (PMC8614338; doi:10.3390/ani11113257)
Supplement: Supplementary file 1 [file animals-11-03257-s001.zip › Table S6.pdf]

Table S6. Contribution of *Prevotella* for other nutrients digestion that was analyzed from metagenomic deep sequencing data of bacteria in Vietnamese goats' rumen.

Enzymes	Number of genes	Enzymes	Number of genes	Enzymes	Number of genes
**1. Esterases**	**3725**	**4. Lipase**	**537**	***-Hemicellulases***	
CE	1621	GDSLlike Lipase	537	GH2	153
Calcineurinlike phosphoesterase	1155	**5. Starch degradation**	**1117**	GH98	131
Thioesterase	332	Alpha amylase	926	GH2-SBD	108
Type I phosphodiesterase	251	GH57	124	GH67	41
AcylACP thioesterase	176	GH66	33	GH115-GH67	35
Esterase like activity of phytase	96	Core2/I Branching enzyme	28	CE2	32
Glycerophosphoryl diester phosphodiesterase	33	GH63	2		
				GH38	30
Esterase PHB depolymerase	32	Alpha amylaseCE	1	GH35	16
Phospholipase/Carboxylesterase	14	GH119	1	GH43-CE	10
protein thioesterase	9	GH126	1	GH10CECE1	9
LigT like Phosphoesterase	5	GH63PE	1	GH2-SBDCE	8
CE-Ig	1	**6. Lignocellulases**	**6199**	GH10CE	7
**2. Glycosyl transferases**	**7153**	GH116	1	GH36	7
GT2	3562	GH118	1	GH28-CE	4
GT1	1925	GH122	1	GH39	3
GT4	429	GH18	47	GH53CE	3
Transglycosylase	396	***- Cellulase***	***699***	GH115-GH20	2
Lipid A disaccharide synthetase	179	GH30	165	GH115-CE1	1
GT11	129	GH124	1	GH115-CE12	1
GT9 (heptosyltransferase)	114	GH128	1	GH115-GH28	1
GT	113	GH16	20	GH115-GH33	1
GT28	82	GH26	164	GH115-GH43	1
GT8	67	GH26AcXE	4	GH117	1
GT90	37	GH3	252	GH120	1
GT25 (LPS biosynthesis protein)	28	GH3-CE	1	GH121	1
GT-CBM	27	GH8	24	GH123	1
GT26	24	GH9	67	GH125	1
GT10 (fucosyltransferase)	15	***-Hemicellulases***	***5344***	GH127	1
GT6	13	GH43	769	GH129	1
GT36	10	GH92	647	GH130	1
GT21	2	GH88	576	GH2-CE	1
Mannosyltransferase	1	GH28	476	GH28-PE	1
**3. Polysaccharide degradation**	**87**	GH10	448	GH43-Lipase	1
Carbohydrate phosphorylase	46	GH115	432	GH43-GT1	1
GH76	34	GH53	387	GH59	1
GH65	4	GH25	301	***-Pretreatments***	***156***
GH85	2	GH20	274	PE	156
GH70	1	GH32	244	**Other**	**6**
		GH31	173	GH56-alpha L fucosyl transferase	6
