# Supplementary figures and images for: Understanding the Role of Prevotella Genus in the Digestion of Lignocellulose and Other Substrates in Vietnamese Native Goats’ Rumen by Metagenomic Deep Sequencing
Source: Animals (Basel). 2021 Nov 14;11(11):3257. doi: 10.3390/ani11113257 (PMC8614338; doi:10.3390/ani11113257)

# KEGG Pathway Classification (sampl1)

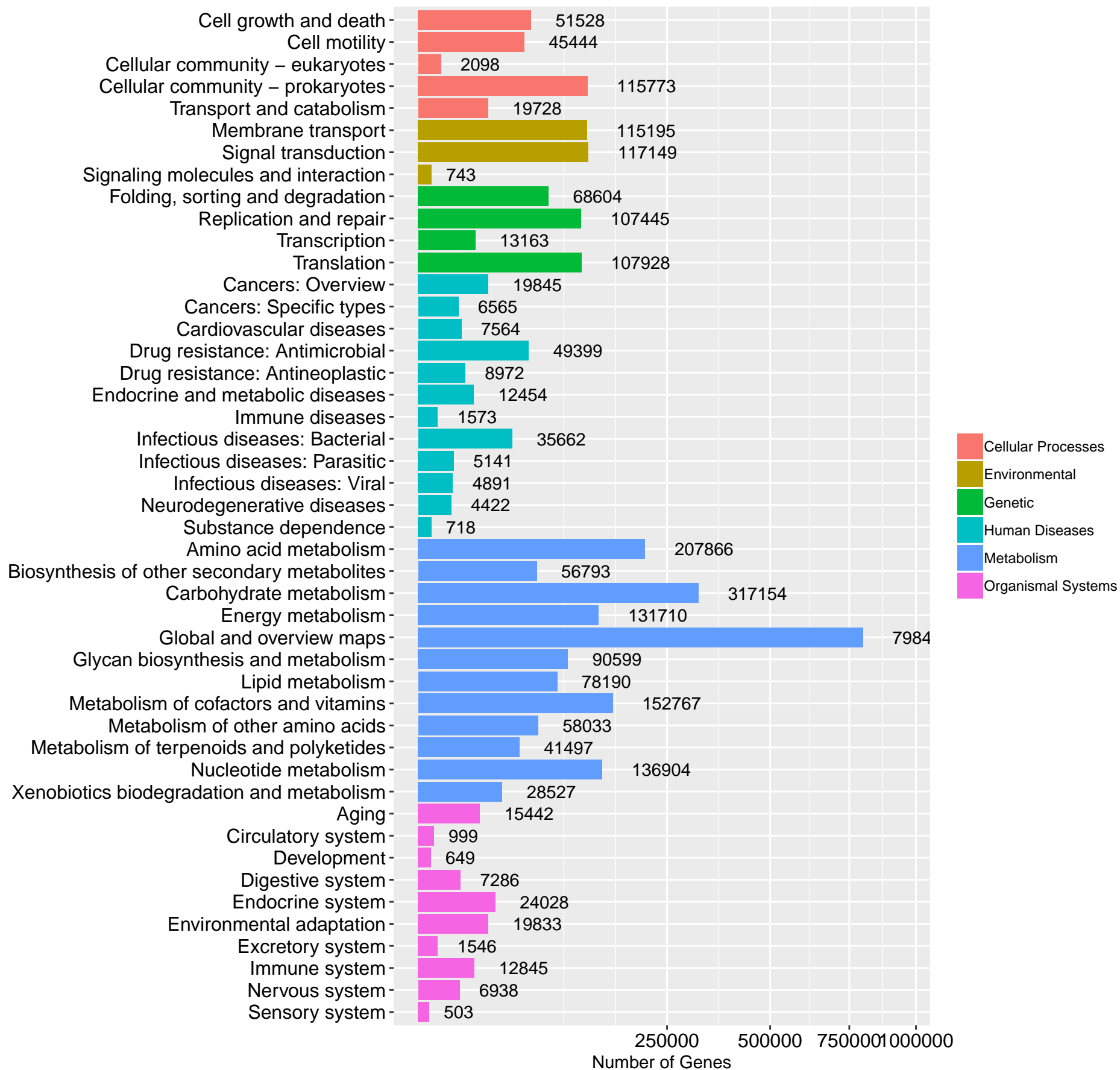

Supplement: Supplementary file 1 [file animals-11-03257-s001.zip › Figure S2 KEGG pathway.pdf]
